# A life course health development model of HIV vulnerabilities and resiliencies in young transgender women in Peru

**DOI:** 10.1186/s41256-023-00317-y

**Published:** 2023-08-21

**Authors:** Sari L. Reisner, Rodrigo A. Aguayo-Romero, Amaya Perez-Brumer, Ximena Salazar, Aron Nunez-Curto, Casey Orozco-Poore, Alfonso Silva-Santisteban

**Affiliations:** 1 Division of General Pediatrics, Boston Children’s Hospital, Boston, MA USA; 2 Division of Endocrinology, Diabetes and Hypertension, Brigham and Women’s Hospital, 221 Longwood Ave, 5th Fl, Boston, MA 02115 USA; 3 Department of Medicine, Harvard Medical School, Boston, MA USA; 4 Department of Epidemiology, Harvard T.H. Chan School of Public Health, Boston, MA USA; 5 The Fenway Institute, Fenway Health, Boston, MA USA; 6 Division of Social and Behavioural Health Sciences, Dalla Lana School of Public Health, University of Toronto Dalla Lana School of Public Health, Toronto, ON Canada; 7 Centro de Investigacion Interdisciplinaria en Sexualidad, Sida y a y Sociedad, Universidad Peruana Cayetano, Lima, Peru

**Keywords:** HIV prevention, Transgender, Latin America, Youth

## Abstract

**Background:**

Globally transgender women (TW) are at heightened vulnerability for HIV infection. In Lima Peru, sharp increases in HIV prevalence are seen among TW ages 25 years and older highlighting the need for early HIV prevention efforts for young TW. This study conducted in-depth qualitative interviews to elucidate the social and developmental contexts of HIV vulnerability for young TW in Lima Peru.

**Methods:**

Between November 2019 and February 2020, young TW ages 16–24 years (n = 21) in Lima Peru were purposively sampled using in-person (e.g., face-to-face outreach) and online (e.g., social media, networking websites) social network-based methods. Interviews were conducted in Spanish and a rapid qualitative analysis was conducted using a modified immersion crystallization methodology to identify themes.

**Results:**

Five themes emerged, informing the conceptualization of a Life Course Health Development Model of HIV Vulnerabilities and Resiliencies: (1) interpersonal contexts (family, school, partners, sexual debut, trans mothers); (2) structural vulnerabilities (poverty, educational constraints, migration, hostile environments, sex work, police violence); (3) concomitant mental health and psychosocial factors (discrimination, violence, depression, suicidality, substance use, life hopes/dreams/future expectations); (4) gender affirmation processes (gender identity development, hormones, surgery, legal name/gender marker change); (5) HIV prevention and treatment barriers (PrEP uptake, HIV care, condom use, risk reduction).

**Conclusions:**

Young TW experience formidable developmental challenges associated with transphobia, violence, and pre-maturely facing accelerated milestones. Developmentally and culturally appropriate interventions to mitigate HIV vulnerability in Peru are needed, including those that consider co-occurring stigma-related conditions in adolescence and young adulthood.

## Background

Globally, transgender women (TW) are at heightened vulnerability for HIV infection with an estimated HIV prevalence of 19.1% (meta-analysis), a 49-fold increased odds of HIV compared to the general population [1]. In Peru, TW are most affected by HIV, with a prevalence of 29.8–48.8% [1–3] compared with 0.2–0.3% in the general population in Lima [4]. An estimated 22,500 TW (95% CI 17,600–27,700) live in Lima, a city with almost 8 million people, where 80% of the country’s HIV cases are concentrated [4]. Young TW ages 16–24 years are a critical group for primary HIV prevention efforts in Peru, due to sharp increases in HIV prevalence among TW ages 25 years and older [3].

Biological risk factors for HIV acquisition and transmission in TW are sexually transmitted infection (STI) co-infections, condomless receptive anal sex, and needle-sharing for injection drugs and gender affirmation hormones and silicone [5–9]. Yet biological HIV risk for TW occurs in the context of widespread transphobia fueling stigma and social exclusion, such as discrimination in employment, sex work for economic survival, and transphobic mistreatment [10, 11]. Pervasive stigma and social exclusion drive HIV disparities for TW [6]. HIV infection is only one of the multiple stigma-related health conditions that TW face [6]. The extent to which young TW in Peru are burdened by stigma and other stigma-related psychosocial conditions such as mental health problems (e.g., depression, hopelessness) and psychosocial risks (e.g., sequelae of psychological trauma, gender-based violence) remains largely unknown and requires additional research [12, 13].

A life course approach can provide insights as to how social identity and position within social structures influence health trajectories in different developmental periods and life stages [14, 15]. A life course model underscores the importance of early life experiences in shaping health, and advances understandings of risks and adverse exposures as occurring across multiple domains, including social, psychological, and structural. Further, health vulnerabilities are conceptualized as co-occurring and clustered based on social inequities, linked to intergenerational exposures, and human agency is situationally-constrained by broader social, cultural, and historical time and place [14, 15]. This approach dovetails with a situated vulnerabilities framework in transgender population health [16]. The HIV vulnerabilities that young TW face are situated—they are not only situated within a social and developmental place or position, but they are also bounded within social institutions that promulgate social and gender norms [16].

Applying concepts from a life course model [15], HIV vulnerability for young TW occurs in a critical period of biological, psychosocial, and cognitive maturation [17]. For young TW, developmentally-linked exposures in late adolescence and young adulthood may uniquely increase risk for HIV infection and other co-infections (e.g., gonorrhea, syphilis). Young TW may face risks for HIV due to being in the developmental milieu of late adolescence and emerging adulthood that their older TW cohort counterparts do not [18]. For example, young TW must navigate developmental tasks common to all youth, such as independence and autonomy [19]. Yet, young TW likely also must contend with tasks specific to being a young TW that cisgender youth do not, and that may increase HIV risk [18]. However, a dearth of research has identified and explored the socio-developmental-specific factors and markers of HIV vulnerability for young TW in Peru. These may include gender dysphoria (e.g., male puberty not aligned with transfeminine or female identity), transgender identity disclosure (e.g., disclosing transgender identity to family, peers, partners), gender minority stress stigma exposures that are youth-specific (e.g., transphobic bullying), sexual objectification in a culture of hegemonic masculine gender socialization (e.g., unwanted sexual advances as transgender women) [20, 21], lack of access to a combination of both youth- and transgender-affirming healthcare (e.g., being referred to by incorrect pronoun or name in HIV/STI prevention and treatment services), and need for uptake of and access to medical gender affirmation (e.g., medically-monitored feminizing hormones). There is a need for research to elucidate the social and developmental contexts of HIV vulnerability in young TW for early HIV prevention efforts and with keen focus on needs outside of resource-rich settings [7].

Furthermore, identifying forms of resistance and enacted resiliencies for young TW across development is paramount for health equity, yet represents a current research gap. In the adolescent health literature, resilience refers to the processes by which youth overcome the adverse effects of risk exposures [22]. Resilience factors that are health-promoting include both individual assets, factors that lie within the individual such as coping skills and self-efficacy, and external resources, factors external to the individual such as parental support, adult mentoring, or community cohesion that focus on socio-environmental context [22]. Discerning how young TW resist the negative effects of risk exposures is important for future programming and services to address stigma-related HIV vulnerability.

This qualitative study explored the social and developmental contexts of HIV risk for young TW ages 16–24 years in Lima, Peru. In addition to vulnerabilities, we also aimed to identify health-promoting factors in youth TW that encourage resilience and resistance in the context of stigma to inform future HIV prevention intervention efforts.

## Methods

### Participants and procedures

This study conducted in-depth qualitative interviews to understand the developmental, mental health, and psychosocial contexts of HIV risk for young TW ages 16–24 years in Peru. Between November 2019 and February 2020, young TW were purposively sampled utilizing a combination of social network-based methods including those conducting in-person (e.g., face-to-face outreach) and online (e.g., social media, social networking websites). Following informed consent procedures, individual interviews were conducted with n = 21 young TW. We began to see redundancy in themes after 15 interviews, and saturation was achieved by interview 21. Interviews were conducted in Spanish by native-Spanish speaking interviewers, audio recorded, and transcribed verbatim. Institutional Review Board approval was obtained from [Universidad Peruana Cayetano Heredia] in August 2019.

### Instrument

A semi-structured qualitative guide was used to assess HIV vulnerability, mental health, and psychosocial contexts in interviews, with an emphasis on identifying developmentally specific exposures for young TW to guide HIV prevention programming and interventions. The qualitative interview guide covered the following topic domains: social context, mental health (psychosocial risks and resilience), sexual behaviors, HIV and AIDS, drug and alcohol use, gender affirming procedures, stigma and discrimination, violence experiences, and social networks.

### Data analysis

We conducted a rapid qualitative approach [23] concurrently collecting data and analyzing it utilizing a modified immersion crystallization method [24, 25]. Drawing from a public health tradition of multidisciplinary inquiry, analyses of transcripts used both inductive and deductive processes to identify themes and relations between themes. One of the key strengths of working across disciplines and geographies is that the team pays keen attention to emergent tensions and contradictions in qualitative data. These points of discord are central to acknowledging the complexity of gender, sexuality, and health across cultures. Immediately following each interview, the interviewer wrote field notes summarizing key themes and findings from the interaction. Next, themes and initial findings were discussed during weekly research team meetings to inform iterative codebook development, achieve consensus on themes, and draw conclusions. Analyses were conducted iteratively such that themes and conclusions were refined by the team with each interview and as data analyses progressed. Transgender community members were included in and consulted about all aspects of study design, recruitment, implementation, analysis, and corroboration of our findings. Further, a constant comparison method [26] of analysis in group-specific differences was used to generate a theoretical model of developmental and psychosocial contexts of HIV risk in young TW. We explored convergence and divergence of themes, and relationships between themes, based on the key characteristics of age and HIV serostatus.

## Results

**Fig. 1:**
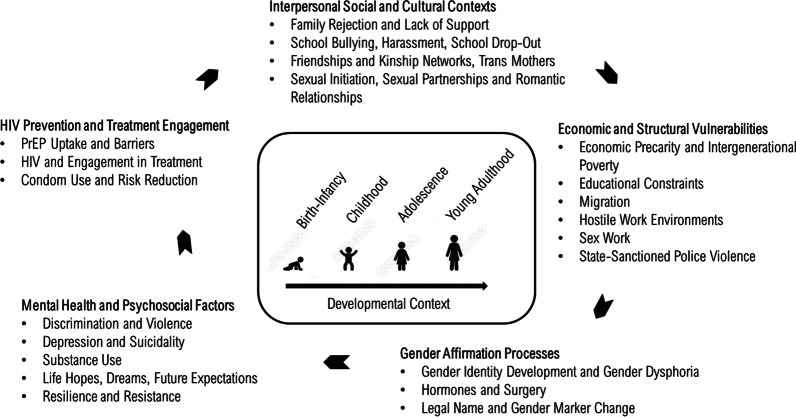
Life course health development model of HIV vulnerabilities and resiliencies among young transgender women in Lima Peru

Mean age of participants was 21 years (standard deviation = 3 years, range 17–24 years). Five thematic groups were identified across qualitative interviews with young TW: (1) social and cultural contexts, (2) socioeconomic and educational factors, (3) mental health and psychosocial wellbeing, (4) gender affirmation processes, and (5) healthcare. Based on these themes and subthemes, we developed a “Life Course Health Development Model of HIV Vulnerabilities and Resiliencies” to understand HIV risk and engagement in HIV prevention and care for young TW. As shown in Fig. 1, this model conceptually situates HIV risk within developmental processes that are dynamic and intersecting with myriad social processes, notably gender affirmation.

### Theme 1: interpersonal social and cultural contexts

Four subthemes were identified within interpersonal social and cultural contexts: family rejection and lack of support; school bullying, teacher harassment and school drop-out; friendships and kinship networks; and sexual initiation, sexual partnerships, and romantic relationships.

#### Family rejection and lack of support

Participants commonly described family rejection and lack of support due to being gay and/or trans. Many described “tense” family relationships. Some reported coming from traditional and conservative families in terms of religious upbringing. Participants reported that family members did not understand their diverse gender identities or expressions and reacted with transphobic physical, psychological, and/or verbal violence. Parents repeatedly insulted them. One 23 year-old participant described her parents who said:

“… [Do not] Behave like that, you’re a man”. But no matter how much they beat me, I was like that, no matter how much they punished me, I kept going. Until I told them ‘that I was not like that [as her parents wanted], that I'm not like that, that I'm not like that.’ They did many things to me, many things ... Until the day that I defined myself, until my mother came here to Lima and said, ‘you are like that’. I already had long hair.

Some participants questioned if their apparent family support was only present because the family was interested in their financial contribution to the household. Others acknowledged that obtaining parental support, including and beyond financial support, was a long process. A 21 year-old participant noted: “My mother and brother wanted to support me even if I’m this way, but when they found out that I changed to a woman, they got worried… they told me they don’t want to see me for now, that they are ashamed because they can’t get used to seeing me this way.”

Many recounted stories of how someone from their family, usually their mother, ended up supporting them in the long run when they had moved and were already living far away. Participants frequently described persistent efforts to form and maintain positive relationships with their families, even after previous emotional and physical abuse. One 23 year-old participant described her father being a supportive parent:

My dad cried, he suffered when I really told him that I am no longer the same as before, that I no longer have male things but things, everything that belongs to a woman… it took my father by surprise, he then cried. Thank God that my father is so wonderful he thanked me for telling him, he supported me, he never turned his back on me, rather my mother was the one who turned her back on me.

#### School bullying, teacher harassment, and school drop-out

Regarding education, most participants (45% n = 9) completed high school, 20% (n = 4) abandoned high school studies, and 24% had middle school education. Six participants (28.6%) had higher education.

Participants reported extreme peer bullying in school (“we are like weirdos”). Most commonly, they recounted insults due to being “effeminate” by peers and felt oppression due to the regulation of gender norms by dress code and expected behaviors within schools. Further, educational institutions were not prepared to support or protect young TW, with teachers sometimes ignoring bullying and/or actively condemning them which further fostered harassment and violence in school.

Participants reported dropping out of school due to consistent and ongoing bullying and harassment. One 23 year-old participant described the mistreatment she experienced and subsequent drop-out: “Yes, I have suffered from bullying…I did not finish my school because of the way they treated me.” Most reported school drop-out between ages 7–14 years.

Despite persistent transphobic bullying in schools, many participants had an enduring desire to continue their education: “It’s really not too late to study…I would love to study, to be back in school, to laugh with my classmates” (23 year-old).

#### Friendships and kinship networks

Most young TW described not having many friends, only acquaintances who they went out with occasionally. As one 23 year-old participant explained: “Girlfriends, girlfriends, I can’t say it like that, because you are a girlfriend only when you help them. I do not consider them girlfriends, more so acquaintances. These, acquaintances, trans girls, I cannot call them friends, because they are not my friends.” Participants described competition between TW. Some participants reported difficulty having real friends in the trans community because they reported other TW as “envious” and “gossipy”. Many did not trust anyone to tell their most intimate things or their problems, and so reported keeping it to themselves.

Many participants reported having at least one friend who supported them, a person generally older and often trans, whom they trusted and from whom they leaned on to receive multiple forms of support. Participants described these “trans mothers” as playing a key role in their lives, older trans women who had survived and overcame struggles of being a trans woman. Trans mothers were described as provided resources including offering stable housing, initiating young TW into sex work, providing advice on hormones including how to procure them and what dosage to take, and demonstrating how to live as trans.

Participants frequently reported mutual aid efforts among communities of TW, such as paying for food or medical bills, even from those who they did not consider friends. One 23 year-old participant summarized this: “I see that all the girls work together, [if] they see a girl who is doing poorly, they support her. [If] she’s sick, if she’s in the hospital they support.” Despite social tensions largely caused by scarcity of resources, TW described how the community supports collective survival.

#### Sexual initiation, sexual partnerships, and romantic relationships

Participants described sexual initiation at early ages and many reported having their first sexual partners at ages 7–8 years. Most recounted their first sexual experiences with older cis men ages 19–40 years, often neighbors, male relatives, friends of their father, teachers, or schoolmates. The majority of participants did not describe their young age of sexual debut as child sexual abuse or sexual assault. Young TW downplayed these experiences. Some described that they were in a situation of perceived autonomy in their lives where they had more “freedom” of gender expression in these dynamics than they had in other contexts, such as at home with their families. Others remarked on the material support (e.g., money, financial support) they gained from these interactions and relationships.

Young TW reported a continued pattern of serious relationships with older men, marked by significant power imbalances particularly regarding age of sexual consent and socioeconomic status. Participants reported frequently meeting cis men sexual partners through online social media and networks. Young TW described seeking “romantic love” and wanting to have a loving and lasting male partner. However, most explained their relationships with men were short and lasted only 3–6 months. They described depression and/or feelings of sadness when these relationships ended, particularly due to the financial burden that had been temporarily removed by the cisgender men who they were sexually active with.

Young TW commonly described seeking gender affirmation from sexual partners. Some reported being afraid of rejection from non-affirming partners. One 18 year-old participant recounted her experience: “Because sometimes I might fall in love with a guy who is gay, not a guy who likes trans girls. And it’s a little difficult, because I am this [trans], I am not a gay boy. Recently it happened to me with a boy, who thought I was gay, but I’m not. And we decided to break up because of that, because he wanted a boy, not a girl.”

Participants reported having suffered physical, sexual, and/or psychological violence and assault from their partners. This intimate partner violence took many forms, including transgender-specific violence comprised of controlling behaviors and psychological abuse tactics that weaponize transphobia within the relationship.

### Theme 2: economic and structural vulnerabilities

Within the theme of economic and structural vulnerabilities, six subthemes emerged: economic precarity and intergenerational poverty; educational constraints; youth migration; hostile work environments; sex work; and state-sanctioned police violence.

#### Economic precarity at a young age and intergenerational poverty in childhood

Most participants reported needing to be financially independent from a young age., For some, this early economic independence was the result of rejection from families and caregivers who rejected them due to their gender identity. Due to this rejection, many young TW were forced to leave the town where they grew up and support themselves. For other participants, economic precarity resulted from intergenerational poverty and having families living in poverty who could not support their educational plans because they did not have the financial means to do so.

Participants described living “day by day”, occupying a precarious economic situation. They commonly describe their housing and living conditions as “unstable”. Most described their current housing as a rented room, often shared with peers and friends, or with a sibling or relative. Young TW reported these supportive relationships were essential to their survival. A few described wanting to pay back those who had helped them, and many had desires to become more independent and rely on less outside support: “I want to study and get ahead on my own, not depend on others to support me” (21 year-old).

#### Educational constraints

Some participants who reported dropping-out of school due to transphobia (see above) expressed interest in resuming education and described moving to Lima to save money. Some were interested in completing high school first and then continuing with higher education in programs such as nursing. Other participants had enrolled in vocational/trade/technical training programs (e.g., to become professional bakers, seamstresses, hairdressers, make-up artists), but in many cases, were unable to finish such programs due to high cost and lack of financial resources. One 17 year-old participant shared why she dropped out: “Because I had no one to support me. Because sometimes my parents, as I have brothers who are also in school and there wasn’t enough money.” Others reported that their families would not support their education because of their trans identity. However, many maintained a resilient desire to learn and continue their education: “I am thinking about working, saving some money. Next year, I’m thinking about studying.” (18 yearold).

#### Youth migration

Migration was common among participants. Some reported internal migration from elsewhere within Peru (e.g., from the jungle, northern Peru). Others reported immigration from outside Peru (e.g., Venezuelan migrants). The majority of participants (86%, n = 18) migrated to Lima from inner cities in Peru and one participant from Venezuela. Most young TW who migrated came to Lima looking for “opportunities”. These opportunities centered on being able to live more freely to safely express themselves in their trans identity and improving their economic prospects. The range of time since migration to Lima was 1–4 years, with most migrating in their late teens. One 21 year-old participant explained her decision to move to Lima as follows:

I also wanted to get out of the jungle because many people talked about me being like this (trans), that was embarrassing to my mother because they always told me even if you are like that, do not behave as you are, behave very manly… That’s why I said, so as not to have more problems for you and so as not to make you more ashamed, I have to get out of here, so I’m not going to bring you shame or you are not going to hear from me…When I finished high school and I quickly left… I started using my woman’s name when I move here to Lima.

#### Hostile work environments

Participants described the developmental process of facing increasingly hostile work environments as they self-actualized and expressed their trans identities outwardly. Participants discussed how their job options were reduced once they expressed their trans identity. Some young TW described they were forced to dress as a man in work environments outside Lima (e.g., in the fields). Inside Lima, young TW started working in restaurants or small shops. Problems arose when they began the process of body modification (e.g., they were allowed to work “as gays” but not “as women”). One 17 year-old participant shared that her employer told her the following: “You’re not going to come here dressed as a woman because it is forbidden…You can dress like that when you are in your room, when you are with your friends, outside of your work you can do it, but when you are at work you cannot.”

Transphobia in the work environment was also discussed in the context of young age. One participant noted that it was especially hard to find jobs being underage and trans: “That’s life here, that is, when they see you like this (visibly transgender), they deny you a job, worse when you’re a minor, they won’t give you one” (17 year-old). Participants explained that a trade-off for coming out as trans and living in the world embodying a female identity was to forfeit the opportunity for gainful employment. Sex work was often described as an option to earn income. For example:

They wouldn’t say anything or told me ‘come tomorrow.’ I would go and ‘no, it's already taken.’ I would go to another restaurant, the same. I got tired and then I got desperate because my money was running out (…) My friends told me: ‘here in Lima, you just won’t find a job, you have to cut your hair, dress as a man. You put on those silicones, it is very difficult to get a job like this.’ I thought, I’m going to try, but the same: ‘come tomorrow’. So… they [her friends] told me, ‘Start working on the street, all the girls start on the street.’ ‘How is that I say?’. ‘Pay for a spot and get to work.’ (23 year-old)

Despite these barriers, many participants reported long-term plans to become financially independent through future employment, and some reported desires to begin a small business. One 19 year-old participant stated: “I want to [find another job], I want to, but I haven’t found the time. Maybe study…maybe hair styling, or skin[care]…cosmetology…I have to know something in life, I don’t just want to survive on prostitution, I’ve had enough.”

#### Sex work

Most participants (86%, n = 18) reported sex work as their main source of income. Participants described that a friend or acquaintance introduced them to street-based sex work. Due to limited job prospects, sex work was frequently described as enacting autonomy and securing needed material resources.

Exploitation in the context of sex work and territorial wars in street-based sex work were commonly reported by participants. Young TW working on the street generally made 30–100 soles (USD $8–$28) per week depending on what their clients requested. Many were required to pay quotas of 20–30 soles (USD $5–$8) to older TW and/or cisgender men (“pimps”) in order to “rent” space on a street corner. Some neighbors contested their presence in the neighborhood, and they were often harassed by police or “serenazgo” (municipal police).

Some participants found clients through social media and online networking sites like Facebook and WhatsApp but they called these “friends” not clients. Other participants noted that they sought clients online during the day and in the street at night. One 18 year-old participant narrated:

I started at 17, publicizing myself (online) but I didn’t go out on the street to be on the corner or stand on the sidewalk… (Initially I didn’t publicize myself) as a trans woman but as a bottom guy that dressed as a girl…I then said, why don’t I publicize myself as a trans woman?... I did so, not only that but I started to go out into the street at night… I may stand outside during the day with little make up or sometimes with just nail polish… but not with a wig… at night like I told you it’s a different production.

Sex work was described as “risky” by participants. Young TW reported negative experiences including assaults with weapons (knives, gun, bottles), robberies, and attempted homicides. Mistreatment was common in sex work encounters, including receiving insults and threats. Young TW reported being made to do things sexually they did not want to do by clients. Many were offered more money by clients to have sex without a condom. While many young TW reported denying this offer where they could, others described the reality of violence impeding their personal agency: “I mean, a guy, I didn’t know he was a madman, and he arrived…I went to attend him, and he got bad…he smoked, did cocaine, and everything, he got mad, ‘close the door’ he said, ‘come on, do me like this, otherwise I’ll stab you, or else I’ll hit you’.” (23 year-old).

Participants reported that “it’s hard” at the beginning when they first began doing sex work, but that later they “toughen up” and it became “normal” to them. This trajectory was particularly noted by participants who were initiated into sex work at younger ages. Most participants reported not liking to work on the street, but they did it “to survive”. Some did it in parallel with other jobs such as being a hairdresser or washing dishes at a restaurant.

#### State-sanctioned police violence

All participants had suffered some form of police violence or harassment, especially in sex work. This state-sanctioned violence was physical, sexual, and psychological in nature. Some participants reported that they knew their rights and that being abused by the police was unlawful, but the majority let it pass as routine in the context of sex work. Many participants reported they had also been violated when they were minors because of their age. Navigating police violence was further complicated for minors and for those who had migrated from outside of Lima from smaller/rural areas with very different levels of police presence. One 23 year-old participant explained: “We live through very difficult circumstances in our lives, sometimes here on the street, more than anything you live a tragedy.” Many participants reported they would never go to the police because they did not trust them and feared additional abuse.

### Theme 3: mental health and psychosocial factors

The theme of mental health and psychosocial factors was comprised of five subthemes: discrimination and violence experiences; depression and suicidality; substance use; life hopes, dreams, and future expectations; and resilience and resistance.

#### Discrimination and violence experiences

Some reported discrimination due to other social statuses besides being transgender. For example, one participant reported discrimination due to being Venezuelan, and especially felt discriminated against by transgender communities. Others reported suffering insults and ridicule (“cabro”, “fagot”) on the street, but all said they ignored it. Some recounted stories of not having been allowed to enter restaurants or hotels. One 18 year-old participant shared her experiences with sex work-related violence: “When I was 17 years old… We got to the hostel and he smoked marijuana. His face totally changed and when I came out of the bathroom, he began to threaten me with a knife. He told me he was going to take my eye out, and he placed the knife there [on her cheek]. Thank God he didn’t do anything to me, but I was shocked… I kept quiet, I told him please don’t do anything to me… because I mean, I didn’t want to die there. And he took my cell phone, my sneakers, my money. And in moments like those, I always see myself in fear.” Participants often described “fear” when narrating personal experiences of pervasive discrimination and violence that occurred as a minor.

#### Depression and suicidality

Participants reported being lonely, needing social support, feeling isolated, and experiencing deep mistrust of peers and social institutions. Most stated that they did not share their problems with others because they did not trust anyone and preferred to keep it to themselves, causing feelings of isolation. All reported periodically feeling sad, frustrated, discouraged, and grieving. Some described crying alone in their rooms. Many did not know where to turn if they had a problem and felt that no one was going to support them in solving their difficulties. Five participants (36%) reported having engaged with mental health counseling or therapy services at some point, and of those who had not, 70% (n = 7) reported wanting to.

Some participants, particularly those who were younger in age, felt they had the capacity to solve their problems alone but often expressed being overwhelmed (“sometimes I feel like I’m going to explode”), highlighting social-emotional and developmental processes of youth agency, coping, and autonomy. Many reported they “get over it” when they distract themselves, hang out with other transgender women and discuss superficial things with them, and wish that “it will pass”. “Having fun” and “not thinking about my problems” were endorsed as coping strategies. One participant had a suicidal episode due to abandonment by a partner.

#### Substance use

Participants reported drinking alcohol “from time to time”, many reported weekly intake of 1–2 drinks in the setting of weekend social outings and holidays. All described having tried cocaine or marijuana but said they do not usually use drugs. Some did not know what other drugs they had used, but knew they had consumed marijuana and cocaine mixed with other substances in the context of sex work. Two young TW reported excess alcohol and drug use to make them forget their problems (“it is good for my head”). For instance, one 19 year-old participant noted: “I smoke marijuana (when I feel sad)… I go out with my friends ‘I'm already sad, what do we do?’ So we go out on the street, we laugh, we listen to music, and we relax… we cook, we go to the market, we go to see movies.” One participant reported drinking in large quantities (“up to a case and a half of beer”) and the another reported using marijuana every day. Alcohol use and marijuana use were described as increased in the setting of sex work, such as bars or clubs.

#### Life hopes, dreams, and future expectations

Participants described future aspirations related to economic security, pursuing lifelong interests, and increased social support. Some wanted to have their own business, especially a hairdressing or beauty salon. Others wanted to dedicate themselves to gastronomy, finish their studies, and have a career, especially in the field of health (nursing or medicine were mentioned). One 21 year-old participant shared: “I moved to Lima to study to get ahead on my own, I want to study. I don’t want to depend on someone else supporting me… So I don’t have to ask my partner that I want this or that… If he leaves, he can leave because I won’t depend on him… If I had a career, I could depend on myself… I will work and I will make it on my own… I would like to (study), well I like cosmetology, I also like cooking. And I like pharmacy.” Autonomy was a goal for many participants. Some saw their future aspirations as achievable, while others saw these as only a dream and not actualizable, regardless of age.

#### Resilience and resistance

In the face of harsh living conditions and experiences of discrimination, stigma, and violence, most participants narrated resilience and resilient-informed action in their desires for a better life. Some sought to make enough money to support their families, others wanted to complete their gender affirmation or transition, and some desired to have influence upon their peers and community. The inner strength of participants was evidenced in sharing testimonies of violence and struggle, and resistance to stigma and oppression from multiple sources. As one participant expressed: “We are stronger than heterosexual people, we live day by day, and every night we have to step outside to live that what gives us life, also gives us cold and hunger. And many have to face their families’ rejection and look for a trans family in this family, and feel more love by other trans girls than from their own families” (23 year-old). Resistance was described as a source of pride and solidarity by many participants, irrespective of age.

### Theme 4: gender affirmation processes

The theme of gender affirmation processes included subthemes for gender identity development and gender dysphoria, hormones and surgery, and legal documentation.

#### Gender identity development and gender dysphoria

Many participants stated that since they were children ages 5–8 years-old they felt like women (they always “felt this way”) based on their gender expression, toys, clothing, games, and attraction to boys. Many described being forced to cut their hair short by their families and at school due to gender norms policing. Some participants moved out or away from family because of and during gender identity development. One 21 year-old participant shared: “Since I was 10 years old, I started wearing a little make-up, but I started dressing as a woman when I was 19 years old… I also started working on the streets at 19… From there I felt everything complete, right? I dressed as a woman, I looked at myself and liked me more and I continued to transform myself more and more and with the instructions that my friends gave me, I took hormones, some hormones and some pills, that is how I dressed as a woman and put on silicone and I’m like this now.” Participants described the developmental arc of recognizing their gender identity as children, experiencing stigma from families and schools (“being trans is bad”) due to their gender, and journeying to accept and embrace their gender identity (“this is my nature”) no matter what the cost of self-actualization.

#### Hormones and surgery

Psychological validation and physical gender affirmation were often linked for participants. Participants typically described the length of time they had been outwardly presenting in a female identity, or had been on hormones, as important milestones in their lives. The majority of the sample (80%, n = 15) reported hormone use. Young TW commonly described finding out about hormones from groups of transgender women. Most participants reported starting hormones at ages 14–16 years and obtaining hormones at pharmacies without prescriptions. No participants reported taking hormones under medical supervision. Lack of medically monitored hormone use (i.e., obtaining blood levels) was attributed to limited medical training and availability of sensitive providers, especially for youth.

Approximately half of participants (53%, n = 10) reported non-medically administered silicone injections (i.e., industrial silicone or “fillers”) to feminize their bodies (e.g., hips, buttocks). Some wanted to be “completely feminine” and were saving money for surgery, particularly facial feminization and breast augmentation. Only one participant reported breast augmentation surgery, but 94% (n = 16) reported wanting to access this procedure. No participants reported orchiectomy or vaginoplasty, though 25% (n = 4) and 21% (n = 3) wanted these procedures, respectively. Other participants were content being trans and inhabiting space across gender categories as nonbinary (“you don’t have to be trans your whole life”). All participants had an ongoing desire and need for gender affirmation.

#### Legal documentation

All participants reported having a national identification document (DNI), but no participants had changed their name nor gone through a process to change their gender on the document. Many participants were uncomfortable with the name on their DNI and wanted to change it. Yet processes of changing the DNI were conditioned by limitations of cost, access to proper legal counseling, and justice system requirements that complicated, inhibited, and/or extended legal processes. Most participants described that having a DNI which did not match their current gender identity or name was a barrier to their accessing healthcare services and education, including HIV prevention and treatment. Some participants were not interested in changing their name on the DNI (“as it is, it’s fine”, “I’m not traumatized in that regard”). Another participant noted: “I feel that is not a priority (to change my name on my DNI)… I don’t care. I would like to, but later on, not now” (18 year-old). Several younger age participants described prioritizing other needs such as their medical gender affirmation and daily survival.

### Theme 5: HIV prevention and treatment engagement

Three subthemes were identified in HIV prevention and treatment: PrEP uptake and barriers, living with HIV and engagement in treatment, and condom use and risk reduction.

#### PrEP uptake and barriers

As context, daily oral PrEP is available at some public primary care facilities in Lima as part of a demonstration study (IMPREP) but not yet integrated within the Peruvian public health system. Two HIV negative interviewees had entered a PrEP program and reported getting regular HIV testing and routine PrEP screening. Several participants knew what PrEP was and had been offered it. Two participants did not want to take PrEP, one because “I do not believe in medications” and the other “because I am taking hormones and do not know if it [oral PrEP] could harm them.” (23 year-old).

One 23 year-old participant shared her mistrust of medical institutions when discussing PrEP: “No, (I will never use PrEP), the World Health Organization is a huge mafia… I have researched a lot on how we are dealing with life systems, politicians only make millionaires richer and people remain their slaves… I have searched for this information online, I’ve seen a lot of information with proof.” Among those who did not know about PrEP, there was interest in learning more about PrEP and potentially taking it to prevent HIV acquisition. Participants did not know whether there were any age restrictions or limitations involved in PrEP care for minors.

#### Living with HIV and engagement in treatment

Although interviewers did not directly ask young TW to report on their HIV serostatus, several participants (n = 5%, 23.8%) ages 17–22 years disclosed living with HIV. These participants reported being on HIV treatment and adhering to antiretroviral medications. One interviewee living with HIV said she was undetectable, but when probed, expressed confusion about what undetectable meant. Only one participant reported feeling that HIV had a negative impact on her mental health. Many continued doing sex work after their HIV diagnosis.

#### Condom use and risk reduction

Most participants reported always using condoms with both sex work clients and friends. Use of lubricant was much less. When asked if she sometimes slipped and didn’t use condoms, one participant responded as follows: “No, some request that they wanted to do it like this (condomless) and I just say no, because I don’t accept it, because I don’t like it, because it’s something weird” (21 year-old). Despite wanting and attempting to always use condoms, HIV risk was introduced when men forcibly sexually assaulted young TW without a condom during sex work. As one participant summarized when asked about her experiences in sex work as a 16 year old: “[You’re] ‘the beaten girl’, when older men want to force you, they force you [to have sex], no? More than anything else…” (19 year-old). Participants also described strategies to reduce risk for other adverse exposures, such as violence, by meeting sex work clients via social media rather than street-based sex work.

## Discussion

Drawing on a life course model of human development [15] and directly learning from the lived-experience of young TW, this study conceptualized a Life Course Health Development Model for HIV Vulnerabilities and Resiliencies in young TW. This model situated vulnerability and resiliency for HIV infection during the developmental milieu of adolescence and young adulthood [17, 22], and for young TW specifically in the context of pervasive societal stigma, discrimination, and transphobic exclusion. Young TW in this study described navigating developmental tasks common to all youth, such as independence and autonomy [19], particularly in relation to romantic relationships and economic independence. Yet, young TW also narrated contending with formidable developmental tasks specific to being a transgender young person. Factors identified by young TW include family rejection, bullying in schools due to breaking gender norms associated with their sex assigned at birth, sexual objectification and exploitation, and childhood sexual assault. Consequently, young TW adolescents were often forced into premature adulthood and contended with developmental tasks well beyond their actual age in years. These experiences provided the backdrop against which HIV acquisition risk occurred, HIV prevention services were delivered, uptake of HIV treatment services was negotiated, and other stigma-related health conditions arose [18].

This study found that young TW were exposed to continuous violence since they became conscious of and expressed their felt gender identity as girls/women. Young TW reported physical, psychological, and/or verbal violence from family members once disclosing a transgender identity. Family rejection posed a major threat for young TW by increasing their potential exposure to economic and structural vulnerabilities such as migration and economic precarity. Transphobic bullying and harassment in educational settings were linked to school drop-out for young TW, increasing risk of poverty and ultimately sex work for economic survival. Violence, mistreatment, and coercion in a sex work context placed young TW at increased vulnerability for HIV, including via economic coercion to not use condoms and subsequent forcible sexual assault. Findings suggest that early life experiences set off cascades and trajectories of linked exposures which increased vulnerabilities for HIV infection in adolescence and young adulthood. Early sexual assault by older men, young age of sexual debut, and patterns of engaging with older cisgender men partners resulted in gendered power dynamics that, when combined with the need for gender affirmation or validation from sexual partners and lack of accessibility or availability of PrEP, was a formula for HIV acquisition risk.

Excluded from school and employment, many young TW in this sample were forced into sex work during childhood and adolescence. Young TW described frequent experiences of sexual abuse, sexual exploitation, and sex trafficking at the hands of older men, and sometimes trans mothers. Minors under the age of 18 engaging in sex work are considered to be victims of the crime of sex trafficking, regardless of the use of force, fraud, or coercion [27]. In addition to heightening HIV vulnerability, sex work among young TW was linked to other adverse health outcomes such as violence, isolation, loneliness, and depression. Results underscore the complex context in which HIV vulnerability occurs for young TW. Young TW were separated from their family of origin due to transphobia, forced into sex work with older men to obtain material support (e.g., money, housing, food), and became members of a new transgender family (especially if connected with a trans mother) drawing on socio-emotional resources of TW community which promote resilience. Young TW often re-contextualized and framed the sexual abuse and trafficking experiences they had in terms of their autonomy—they described exercising their own autonomy to express their female gender identity and have the financial means to become themselves. Together these study findings highlighted that young TW were forced to find gender affirmation in developmentally inappropriate and dangerous spaces, such as sex work because they were denied familial, educational, and workplace opportunities.

Additionally, this study found that developmental processes for young TW pertained not just to their age, as common for all youth, but also in relation to their gender as transgender young people. Gender affirmation processes, including age of outward presentation in a female identity and first hormone use, were described as developmental milestones by young TW in this study. Yet outward expression of womanhood not only demarcated living authentically, but also onset of extreme stigma-related struggles such as difficulty with employment. For many, pre-mature exposure to developmental tasks resulted from family rejection and leaving their families and hometown to migrate to Lima, in several cases still as minors and not at the age of majority. Thus, young TW were often prematurely tasked with navigating adulthood as adolescents. Further, current age in years for young TW was often different than their social age as women, based on the length of time they outwardly presented in a female identity or were on hormones. It is important to consider the developmental period that young TW are in for their actual age (e.g., emerging adulthood) as well as the social age they occupy as young TW (e.g., length of time living in their gender identity as women) in understanding HIV risk and engagement in HIV prevention and treatment. Young TW prematurely reached young adulthood and faced developmental experiences in adolescence that would typically occur in young to middle adulthood.

Our results indicated that the trans community plays an important yet complex role in the lives of young TW. Young TW had some mistrust of trans community, especially insofar as they reported withholding more personal and intimate thoughts or problems to avoid gossip. But there was also a sense of community cohesion and culture reinforced by shared marginalization and experiences as TW. This was seen in the giving and receiving of horizontal mutual aid such as helping with food and medical bills even when TW did not consider themselves friends. Further, this studya demonstrated the pivotal role of trans mothers in the lives of young TW, elders who had survived and learned to live with the stigma of being a trans woman, highlighting the close community networks and ties between younger and older TW. Trans mothers provided tangible resources and support to help young TW, shaping behavioral norms within the community, an example of external resilience resources that young TW accessed [22]. Yet, consistent with prior research findings [28], the exploitative nature of how support may be provided by and for TW necessitates consideration. One example is the way that trans mothers stopped engaging in sex work and instead provided facilities for a fee from their young TW daughters to engage in sex work. We identified complex intergenerational dynamics at play within the trans community that warrant further research and attention in future intervention efforts. The intergenerational exploitative cycle of sex work resulted from a failure by the State to provide viable employment options to trans women in Peru. The cycle will continue until structural changes take place that address the employment barriers faced by young TW and offers alternatives such as the transgender job quota law in Argentina. Discerning how young TW resist the negative effects of risk exposures is important for future programming and services to address stigma-related HIV vulnerability.

Young TW in this study discussed mental health morbidity, including depression, loneliness, feelings of social isolation, and substance use, yet few participants reported accessing mental healthcare. Mental health and psychosocial conditions commonly onset and occur in adolescence and young adulthood [19]. In the context of gender-related oppression facing young TW, it is possible that these conditions may arise earlier in life, with greater severity, and have lasting and life-long effects, including increased probability of re-occurrence. Further, mental health and psychosocial vulnerabilities may co-occur and synergistically potentiate risk for HIV in young TW [29] as well as influence biobehavioral HIV prevention targets such as sex with condoms, sterile needle use, and PrEP indications [12]. As elucidated by the current study, young TW communities face stigma in healthcare, including due to inability to change name or gender identity on DNI documents required to receive care as elucidated, non-coverage of gender transition procedures, and untrained providers. Corroborating prior research, our study found that young TW without safe and affordable access to youth- and trans-affirming medical services sought medical gender affirmation through unsafe channels [30] (e.g., non-prescribed or medically unmonitored hormones and/or silicone use fillers for feminization) [2, 31, 32]. HIV vulnerability may include sharing needles for hormones and/or silicone. It will be critical to quantitatively characterize the unique concomitant mental health and psychosocial vulnerabilities driving HIV vulnerability in the context of stigma in healthcare for young TW to design and inform interventions that effectively mitigate these risks. Interventions targeting developmentally specific clusters of stigma-related co-morbidities may be of particular interest.

Alongside vulnerabilities, this study highlighted resiliencies such as gender affirmation [33, 34]. Consistent with adolescent research that resilience includes both individual assets and external resources [22], young TW in this sample exhibited resilience via health-promoting individual factors (e.g., coping) and external resources (e.g., material support from community). To foster active engagement in biobehavioral HIV prevention for young TW, it will be vital to leverage factors that promote HIV-related resiliencies in the face of pervasive stigma [22, 35]. HIV prevention efforts continue to largely focus on risk-based approaches in Peru, yet gender-affirming strengths-based approaches are needed which harness community [11, 33] to engage young TW in HIV prevention and care services [7]. For young TW, HIV prevention using strengths-based strategies that are adapted to their youth experiences are necessary [12]. This study found young TW resisted stigma and oppression from multiple sources, demonstrating the potential of this population to persist and resist stressors, evidence of resilience that needs to be re-enforced and built upon for HIV prevention and treatment efforts.

This study has several limitations. Findings are not representative due to the purposive convenience sampling method. We lacked biological confirmation of HIV serostatus. Social desirability bias due to face-to-face interviewing may have resulted in underreporting of HIV risk behaviors, such as condomless sex. This was a small sample size, though appropriate for qualitative research given that redundancy and saturation were achieved.

## Conclusions

Reducing HIV disparities for TW globally will require early HIV prevention efforts, including those targeting social and developmental risks uniquely occurring during adolescence and young adulthood for young TW. Data from this study and the conceptual model that emerged from interviews with young TW can be used to inform developmentally and culturally appropriate interventions for this population in Peru. To maximize impact on the HIV epidemic and decrease the HIV burden for TW, strategies are needed that are capable of effectively mitigating HIV vulnerability among young TW. Young TW experience formidable developmental challenges associated with transphobia, violence, and pre-maturely facing accelerated milestones. Interventions that are gender-affirming, strengths-based, target co-occurring stigma-related conditions, and respond to the unique socio-developmental contexts in which HIV vulnerability occurs represent an important next step to curb the HIV epidemic in this population.

## Data Availability

Contact the corresponding author for access to data and materials.

## Abbreviation

TW: Transgender women
